# Seronegative Bilateral Pulmonary Hydatid Cysts in a 15-Year-Old Boy From Pakistan: Report of a Rare Case

**DOI:** 10.7759/cureus.57958

**Published:** 2024-04-10

**Authors:** Rizwanullah, Yoalkris E Salcedo, Vinoothna Reddy Kayeetha, Mudassir Shah, Syed Owais Haseeb

**Affiliations:** 1 Internal Medicine, Hayatabad Medical Complex Peshawar, Peshawar, PAK; 2 Surgery, Universidad Iberoamericana, Dominican Republic, DOM; 3 Medicine and Surgery, Mamata Medical College, Khammam, IND; 4 Pediatric Medicine, Hayatabad Medical Complex Peshawar, Peshawar, PAK

**Keywords:** pulmonary echinococcosis, surgical excision, albendazole therapy, echinococcus granulosus, case report, pakistan, 15-year-old boy, bilateral, pulmonary hydatid cysts, seronegative

## Abstract

Hydatid disease, attributed to the tapeworm *Echinococcus granulosus*, poses a significant health threat in regions where it is endemic. Here, we present a case involving a 15-year-old boy from rural Pakistan who initially sought medical attention due to a persistent cough and hemoptysis. Despite initially testing negative for serological markers, imaging studies revealed well-defined cysts in both lungs. Confirmation of the diagnosis was achieved through histopathological examination. Management includes albendazole therapy and surgical excision of the cyst. Our case underscores the diagnostic challenges associated with seronegative cases and underscores the importance of considering hydatid disease in endemic regions, irrespective of typical serological markers. This report enhances understanding regarding the clinical presentation, diagnostic approach, and management strategies for pulmonary hydatid cysts.

## Introduction

Hydatid disease, a parasitic infection transmitted from animals to humans, is caused by tapeworms, notably *Echinococcus granulosus*. This parasite's life cycle involves both definitive and intermediate hosts. Adult tapeworms inhabit the small intestines of dogs or other carnivores, and their eggs are expelled through feces. Once ingested by an intermediate host, typically sheep, the eggs undergo development into hexacanth embryos within the liver through the portal circulation. Subsequently, these embryos give rise to cysts within the liver. Unbeknownst to humans, they become intermediate hosts by consuming water or vegetables contaminated with echinococcal eggs [1].

Echinococcal cysts primarily target the liver, representing over 65% of cases, followed by the lungs, which account for 25%. Occurrence in other organs such as the spleen, kidneys, heart, bones, and central nervous system is infrequent. The slow growth rate of these cysts contributes to their late onset in adulthood, often emerging long after the initial infection was acquired during childhood [2]. The symptoms commonly linked with a pulmonary hydatid cyst (PHC) typically begin with cough as the primary complaint, followed by manifestations such as chest pain, breathlessness, expectoration, fever, hemoptysis, and occasional episodes of anaphylactic phenomena [3]. In this report, we outline the case of a 15-year-old boy who initially sought medical attention due to cough and hemoptysis. Upon further investigation, it was discovered that he had isolated bilateral pulmonary hydatid cysts, despite initially testing negative for serological markers.

## Case presentation

A 15-year-old adolescent from a rural region of Pakistan presented with a two-month duration of progressively worsening dyspnea and cough. Initially dry, the cough had recently transitioned to producing blood-stained sputum in the week leading up to admission. Despite lacking any significant medical history, the boy exhibited weight loss during the illness and experienced intermittent low-grade fevers. There was no reported exposure to tuberculosis. Hailing from a farming household with livestock, the boy appeared moderately pale during examination, though not cyanotic, with oxygen saturation at 97% on room air. His pulse rate measured 84 beats per minute, his blood pressure was 120/70 mmHg, and he maintained a normal body temperature of 36.7°C.

Chest examination showed bronchial breathing and fine crackles in the right middle zone and left lower lobe along with dull percussion bilaterally. No peripheral lymphadenopathy was appreciated. Other examination findings were unremarkable. His routine investigations showed normal renal function tests (RFT); liver function tests (LFT), and lactate dehydrogenase (LDH). Gene experts and sputum acid-fast bacilli (ABF) for *Mycobacterium tuberculosis* were negative. The rest of the laboratory investigation is given in Table 1).

**Table 1:** Lab investigations during hospital stay McL: microliter, g/dL: gram/deciliter,  fL: femtoliter, %: Percent, mg/dL: milligram per deciliter, mm/hour: millimeter per hour, ng/mL: nanogram per milliliter

Investigations	Reference range	Results
White cell count (x10^3^/mcL)	4-11	13
Hemoglobin (g/dL)	11.5-17.5	11.8
Platelet count (x10^3^/mcL)	150-450	167
Mean Corpuscular volume(fL)	76-96	82.4
Hematocrit (%)	36-54	38
Neutrophils (%)	40-60	57
Lymphocytes (%)	20-40	33
Monocytes (%)	2-8	4
Eosinophilia of (%)	1-4	5
CRP(mg/dL)	<0.5	2.36
ESR(mm/hour)	0-15	35
Ferritin(ng/mL)	13-150	287

**Figure 1:**
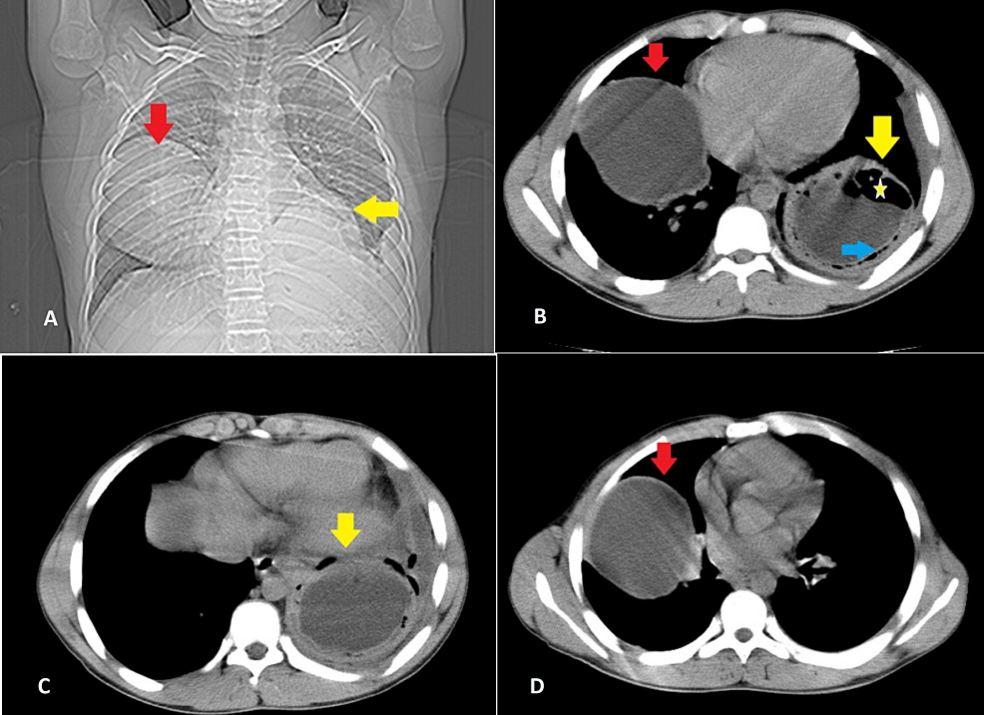
CT Chest CT chest revealed an approximately 8 × 7 cm well-defined cyst in the right mid-zone (panels A, B, and D (red arrow)). Another approximately 8 × 7 cm thick-walled cyst was observed in the left lower zone (panels A, B, and C (yellow arrow)) with an air bubble (panel B, yellow star) and a detached wall (panel B, blue arrow).

As our X-ray machine was out of order, we did an ultrasound abdomen and chest which showed a well-circumscribed bilayer cystic mass on the left lower lobe with a volume of 215 ml with evidence of internal septations and another lesion on the right mid-zone with almost the same volume. Ultrasound abdomen was unremarkable. A chest computed tomographic (CT) scan revealed an about 8 × 7 cm, well-defined cyst in the right mid zone with patchy underlying lung collapse, and no evidence of pleural thickening or pleural effusion. An about 8 × 7 cm thick-walled cyst with an air bubble was observed in the left lower zone with minimal pleural effusion, no evidence of lymphadenopathy, and mediastinum displacement (Figure 1).

CT abdomen and pelvis was normal. After excluding tuberculosis we had a high suspicion of pulmonary hydatid cyst considering his history and imaging findings, we sent for *Echinococcus granulosis* serology by enzyme-linked immunosorbent assay (ELISA), which came out to be negative.

**Figure 2:**
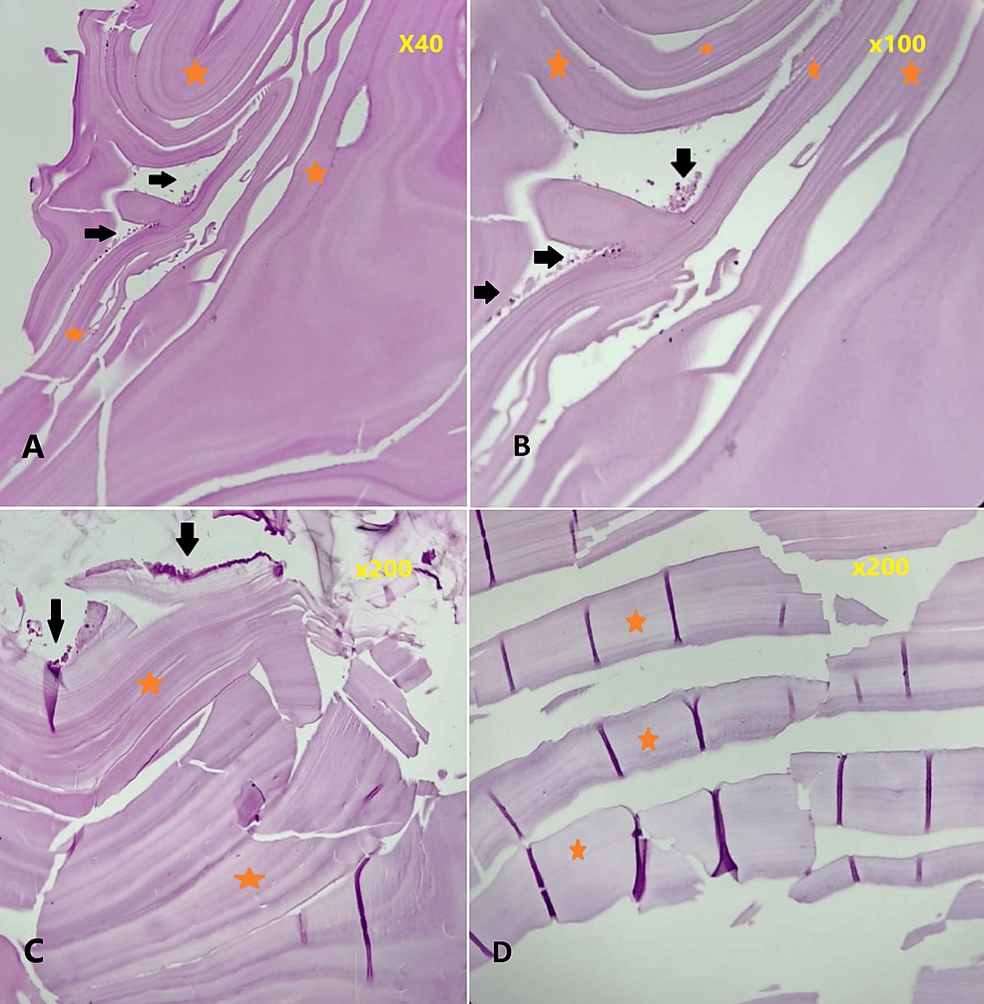
Histopathology, hematoxylin and eosin stain images of hydatid cyst. The magnification of each image is mentioned at the top right corner. The figure shows lamellated cyst walls in panels A, B, C,  and D (red stars) with scattered neutrophils, eosinophils, and evidence of hemorrhage in panels A, B, and C (black arrows).

We started the patient on the standard regimen of albendazole (ABZ) of 28 days cycle with a break of 14 days between courses. Given at a dose of 10-15 mg/kg/day (patient weight 40 kg) in two divided doses. We referred this case to a cardiothoracic surgeon. They excised only the cyst on the left side, but it ruptured during handling after complete removal. On gross examination, the empty cyst appeared as a pearly white cyst wall. We submitted this specimen for a histopathology report, which revealed a lamellated cyst wall with scattered neutrophils, eosinophils, and evidence of hemorrhage (Figure 2).

The patient's contact history, eosinophilia, imaging results, and histopathological findings collectively confirmed the diagnosis of seronegative isolated bilateral pulmonary hydatid cysts.

## Discussion

Hydatid cyst disease, resulting from *Echinococcus granulosus* infection, is widespread in specific developing regions. While it commonly targets the liver and lungs, it can manifest in any organ. Individuals of all age groups, including children, adolescents, and adults, are susceptible to this condition. Notably, our case, like numerous others, demonstrates a male preponderance, likely due to the frequent interaction between boys and dogs, as observed in multiple studies [4]. Echinococcal cysts predominantly affect the liver, constituting more than 65% of instances, with the lungs being the second most common site, comprising 25% of cases. Involvement of other organs such as the spleen, kidneys, heart, bones, and central nervous system is rare. The delayed onset of symptoms in adulthood, often occurring years after the initial childhood infection, is attributed to the slow growth rate of these cysts [2]. Symptoms frequently associated with a pulmonary hydatid cyst (PHC) usually commence with coughing as the primary symptom, followed by indications such as chest pain, difficulty breathing, coughing up phlegm, fever, coughing up blood, and sporadic occurrences of anaphylactic reactions [3].

Typically, cysts larger than 5 cm in diameter lead to bronchial compression. Common complications associated with pulmonary hydatid cysts include cyst rupture, secondary infection, suppuration, and pneumothorax. When the cyst ruptures, it can trigger sudden symptoms such as chest pain, coughing up blood, cough, and fever, or sometimes even a salty taste in the mouth. Hypersensitivity reactions may also occur due to the ruptured cyst, ranging from hives and wheezing to potentially life-threatening anaphylaxis, thereby posing a significant risk to the individual's health [5]. Our case initially manifested with cough and hemoptysis without any complications, subsequently diagnosed as isolated pulmonary hydatid cysts. Screening for hepatic hydatid cysts is recommended for all patients with pulmonary hydatid cysts (PHCs) due to the frequent coexistence of these conditions and the often asymptomatic nature of cysts in the liver. Studies have shown that patients with hepato-PHCs tend to present earlier than those with PHCs alone [6].

In endemic regions, diagnosis is often straightforward based on patient history and radiological examinations, as was the case with our patient, where ultrasound and CT scan findings strongly indicated the diagnosis. Additional diagnostic tests such as immunoelectrophoresis and histopathology, along with enzyme-linked immunosorbent assay (ELISA), are utilized for both diagnostic confirmation and screening purposes [7]. The radiological appearance of pulmonary hydatid cysts can vary depending on whether complications are present. These cysts are typically classified into two groups: uncomplicated and complicated. Complicated cases may involve scenarios such as contained rupture, complete rupture, or superinfection. Uncomplicated pulmonary hydatid cysts typically present as well-defined lesions with smooth walls of varying thickness. CT density measurements often reveal low Hounsfield unit (HU) values, indicating fluid content. Centrally located cysts tend to be round, whereas those situated peripherally may exhibit oval or polycyclic shapes. Daughter cysts and calcifications are rarely observed in pulmonary hydatid cysts. On the other hand, complicated cysts are characterized by rupture and/or infection, with ruptures occurring in up to 47.5% of cases [8].

Similar to our case, a contained rupture happens when the pericyst separates from the endocyst. Due to the encasement of the cyst contents by the pericyst, the probability of a contained rupture causing allergic reactions or infection is minimal. Imaging features of a contained rupture comprise the air crescent sign, inverse crescent sign, and air bubble sign [9]. In our particular case, the CT scan of the chest revealed an uncomplicated cyst on the right side and a complicated cyst on the left side, characterized by the presence of the air crescent sign and air bubble signs. The primary treatment for PHCs typically involves surgical intervention, which is considered the preferred approach. In some cases, pharmacological treatment may be utilized alongside or as an alternative to surgery [3]. Pharmacological treatment involves oral administration of benzimidazole drugs such as mebendazole or albendazole. This approach is particularly considered for managing smaller cysts, patients for whom surgery is contraindicated, those with disseminated disease or multiple and recurrent cysts, and individuals who experience intraoperative spillage of hydatid fluid.

Albendazole is the preferred medication for treating pulmonary hydatid cysts (PHCs). The typical recommended dosage is 10-15 mg/kg/day, taken twice daily, for duration of at least 3-6 months. Continuous administration of the medication has been shown to be more effective compared to the previously believed method of interrupted monthly dosages with a 2-week gap to prevent hepatotoxicity [10]. As a common practice in our setup, we started the patient on an interrupted regime of albendazole and referred this case to a cardiothoracic surgeon, who removed the left-sided complicated cyst with no complications during surgery.

## Conclusions

In conclusion, the presented case highlights the diagnostic and therapeutic challenges posed by isolated bilateral pulmonary hydatid cysts, particularly in regions endemic to *Echinococcus granulosus* infection. Despite initial negative serological markers, the patient's clinical presentation, imaging findings, and histopathological evidence confirmed the diagnosis, underscoring the importance of a multifaceted diagnostic approach. Furthermore, the case emphasizes the need for a high index of suspicion, especially in individuals with relevant exposure history and characteristic symptoms such as cough and hemoptysis. Treatment involved a combination of pharmacological therapy with albendazole and surgical intervention, showcasing a comprehensive management strategy for this intricate condition. This report not only sheds light on the intricacies of pulmonary hydatid cyst disease but also underscores the significance of timely diagnosis and appropriate management to mitigate potential complications and improve patient outcomes.
